# Restoration of *Lactobacillus johnsonii* and *Enterococcus faecalis* Caused the Elimination of *Tritrichomonas* sp. in a Model of Antibiotic-Induced Dysbiosis

**DOI:** 10.3390/ijms25105090

**Published:** 2024-05-07

**Authors:** Yulia Makusheva, Elena Goncharova, Victoria Bets, Anastasya Korel, Elena Arzhanova, Ekaterina Litvinova

**Affiliations:** 1 Faculty of Physical Engineering, Novosibirsk State Technical University, 630073 Novosibirsk, Russia; ys.makusheva@gmail.com (Y.M.); goncharova-ep@rambler.ru (E.G.); v.becz@corp.nstu.ru (V.B.); akorel@gmail.com (A.K.); 2 Department of Natural Sciences, Novosibirsk State University, 630090 Novosibirsk, Russia; e.arzhanova@g.nsu.ru

**Keywords:** microbiota, IBD, antibiotic treatment, bacteria recovery, protozoa

## Abstract

Inflammatory bowel disease (IBD) is a multifactorial disease involving the interaction of the gut microbiota, genes, host immunity, and environmental factors. Dysbiosis in IBD is associated with pathobiont proliferation, so targeted antibiotic therapy is a rational strategy. When restoring the microbiota with probiotics, it is necessary to take into account the mutual influence of co-cultivated microorganisms, as the microbiota is a dynamic community of species that mediates homeostasis and physiological processes in the intestine. The aim of our study was to investigate the recovery efficacy of two potential probiotic bacteria, *L. johnsonii* and *E. faecalis*, in *Muc2*^−/−^ mice with impaired mucosal layer. Two approaches were used to determine the efficacy of probiotic supplementation in mice with dysbiosis caused by mucin-2 deficiency: bacterial seeding on selective media and real-time PCR analysis. The recovery time and the type of probiotic bacteria relocated affected only the number of *E. faecalis*. A significant positive correlation was found between colony-forming unit (CFU) and the amount of *E. faecalis* DNA in the group that was replanted with probiotic *E. faecalis*. As for *L. johnsonii*, it could be restored to its original level even without any additional bacteria supplementation after two weeks. Interestingly, the treatment of mice with *L. johnsonii* caused a decrease in the amount of *E. faecalis*. Furthermore, either *L. johnsonii* or *E. faecalis* treatment eliminated protozoan overgrowth caused by antibiotic administration.

## 1. Introduction

The gut microbiota has a significant impact on the pathogenesis and complications of inflammatory bowel disease (IBD) in humans, namely Crohn’s disease and ulcerative colitis. Chronic inflammation in these conditions arises primarily due to an imbalance in microbiota and the activation of immune response to intestinal bacteria. Some pathogens, including highly virulent strains of *Escherichia coli*, *Bacteroides* spp., *Mycobacterium avium subspecies paratuberculosis*, and intestinal protozoa, can exacerbate inflammatory–necrotic processes in the intestinal mucosa of individuals with IBD [1]. Antibiotic therapy is essential to the treatment of these illnesses, as it diminishes the number of bacteria in the intestinal lumen and alters the composition of the intestinal microbiota. Unfortunately, the administration of most antibiotics diminishes the overall bacterial diversity, suppressing not only pathogenic bacteria but also commensal bacteria, thereby leading to the proliferation of drug-resistant bacteria (e.g., *Clostridium difficile*), fungi (*Candida*), and bacteriophages [2].

Furthermore, certain protozoa possess antibiotic resistance and can inhabit vacant bacterial niches [3,4]. For instance, a decline in bacterial numbers corresponded with an elevation in *Tritrichomonas* sp. DNA in the colon [5]. Currently, intestinal trichomonas virulence is explained by its increased adhesion to host intestinal epithelial cells [6]. Recently, it was shown that trichomonas-derived microvesicles alter the metabolism of epithelial cells, increasing the sensitivity of the cells to trophozoites (an actively reproducing stage of the protozoan life cycle) [7]. Interestingly, *Tritrichomonas* infection caused only mild changes in the histopathology of intestinal tissue while promoting the development of colitis and colorectal tumors [8]. In general, *Tritrichomonas* rarely infect humans, with only several cases reported for immunosuppressed patients [9]. However, there is evidence that trichomonads possess zoonotic potential [10]. Furthermore, research on *Tritrichomonas* may provide data about its human ortholog, *Dientamoeba fragilis*, which is known to have an influence on IBD [8]. Currently, the scientific community is searching for new approaches to the treatment of protozoan infections.

Probiotics are live microorganisms, bacteria, or yeasts that have beneficial effects on health. Some probiotic strains, such as *Bifidobacterium* and *Lactobacillus*, are used in the treatment of IBD. These strains were isolated from limited sources, mostly including fermented milk products. Recently, various candidate microorganisms isolated from the microbiota of healthy humans have been investigated for use as probiotics: *Clostridium*, *Firmicutes* spores, *Bacteroides*, and *Roseburia* [3,11].

In our work, we investigated the efficacy of microbiota recovery after antibiotic administration in mice with a disruption of the *Muc2* gene. The *Muc2* gene encodes the protein mucin 2, which is involved in the formation of the protective mucin layer in the small and large intestines, and disruptions of this gene lead to a significant decrease in mucin synthesis [12]. Furthermore, *Muc2* deficient mice demonstrate disturbance of microbiota composition [13,14]. It is known that hallmarks of IBDs are the thinning of the protective intestinal mucin layer and alteration of the intestinal microflora [15]; therefore, *Muc2*^−/−^ mice are an adequate model of IBD. In our study, we used strains of *Lactobacillus johnsonii* and *Enterococcus faecalis* isolated from feces of healthy specific-pathogen-free-(SPF)-status mice as potential probiotic therapy of antibiotic-treated mice. In addition to bacterial dysbiosis, the antibiotic treatment of *Muc2*^−/−^ mice led to the induced growth of intestinal protozoa such as *Tritrichomonas* sp. [5]. This effect can be explained by the resistance of protozoa to antibiotics and reduced competition for energy resources due to bacteria depletion. Importantly, prolonged antibiotic therapy of *Muc2*^−/−^ mice resulted in animal death and the DNA of protozoa *Tritrichomonas* sp. was isolated from cecal contents [5].

The standard method for investigation of human microbiota composition is the bacterial culture, which allows for the estimation of the number of viable microorganisms. However, it is believed that microbiological methods do not fully reflect the real state of intestinal microflora as up to 80% of intestinal microbiota microorganisms are difficult to culture and thus cannot be detected by microbiological methods [16]. Recently, molecular diagnostic methods have been actively developed and introduced for qualitative and quantitative assessment of intestinal microflora. Real-time PCR analysis is actively used not only in various experimental studies but also in clinical practice, in particular, when studying the effect of probiotic strains on the human microbiota. However, PCR analysis relies on total bacterial content and cannot provide an estimation of living bacteria amount. In this study, we compared the recovery rate of two bacterial strains gavaged to *Muc2*^−/−^ mice after an antibiotics course using two methods: PCR assay and bacterial culture on selective media.

## 2. Results

Two probiotic strains, *L. johnsonii* and *E. faecalis*, were selected to analyze the recovery of the gut microbiota of *Muc2*^−/−^ mice after antibiotic administration. The *L. johnsonii* strain was isolated from the feces of healthy C57BL/6 mice with normal microbiota. The *E. faecalis* strain was isolated from the feces of *Muc2*^−/−^ mice. Microbiological analysis showed phenotypic homogeneity of the isolated strains. Confirmation of the identity of the isolated strains was performed based on the analysis of single colonies of bacterial culture by 16S rRNA gene sequencing (Table 1).

Analysis of *L. johnsonii* and *E. faecalis* content in *Muc2*^−/−^ mice feces in steady state by bacterial culture showed that the number of bacteria of these species differs more than 50 times. Thus, the amount of *L. johnsonii* was 1521.94 × 10^7^ CFU/g feces, and *E. faecalis* was 2890.95 × 10^5^ CFU/g feces (Mann–Whitney Z = 2.3 *p* = 0.02). Analysis of bacterial DNA by real-time PCR revealed similar differences. The amount of *L. johnsonii* DNA was 15,118.45 × 10^−3^ conventional units (c.u.) relative to total bacterial DNA (16S rRNA gene), while *E. faecalis* DNA amount was 3384.35 × 10^−3^ c.u. (Mann–Whitney Z = 2.1 *p* = 0.04).

The application of broad-spectrum antibiotics for one week resulted in the complete absence of alive *E. faecalis* in the feces of *Muc2*^−/−^ mice, whereas *L. johnsonii* was detected in the feces, and the number of colonies was 9271.62 × 10^3^ CFU/g feces. Only after 2 weeks of antibiotic treatment did the number of *L. johnsonii* colonies decrease to 1–2 CFU/g feces. Thus, *E. faecalis* was more sensitive to the antibiotics applied and completely disappeared after one week. While *L. johnsonii* was relatively more resistant and, although decreased, was still presented in samples after 2 weeks of antibiotic administration. In order to stimulate microbiota recovery after administration of antibiotics for 2 weeks, we used three treatment schemes: the first group of mice were left without any treatment, and the second and third groups of mice were gavaged with either *E. faecalis* or *L. johnsonii* strains, respectively.

**Figure 1:**
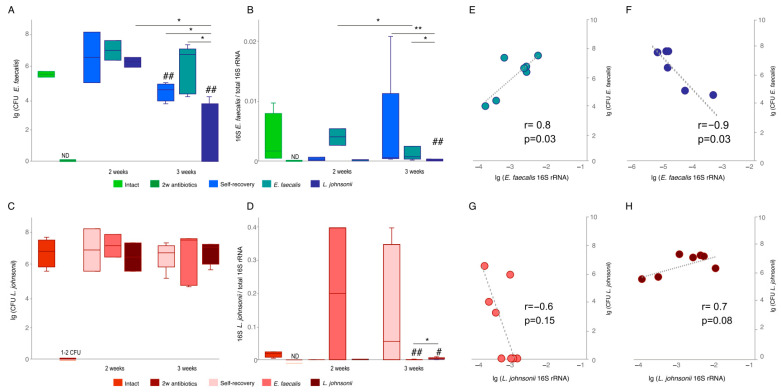
CFU and 16S rRNA of *E. faecalis* and *L. johnsonii* in mice after self-recovery or feeding with *E. faecalis* and *L. johnsonii*. (**A**). CFU of *E. faecalis* in mice feces after 2 and 3 weeks of self-recovery or feeding with *E. faecalis* and *L. johnsonii*; (**B**). 16S rRNA of *E. faecalis* to total 16S rRNA in feces of mice after 2 and 3 weeks of self-recovery or feeding with *E. faecalis* and *L. johnsonii*; (**C**). CFU of *L. johnsonii* in feces of mice after 2 and 3 weeks of self-recovery or feeding with *E. faecalis* and *L. johnsonii*; (**D**). 16S rRNA of *L. johnsonii* to total 16S rRNA in feces of mice after 2 and 3 weeks of self-recovery or feeding with *E. faecalis* and *L. johnsonii*; (**E**). Correlation of CFU and 16S rRNA of *E. faecalis* in feces of mice fed with *E. faecalis*; **F.** Correlation of CFU and 16S rRNA of *E. faecalis* in feces of mice fed with *L. johnsonii*; (**G**). Correlation of CFU and 16S rRNA of *L. johnsonii* in feces of mice fed with *E. faecalis*; (**H**). Correlation of CFU and 16S rRNA of *L. johnsonii* in feces of mice fed with *L. johnsonii*. *—*p* < 0.05 and **—*p* < 0.001, PERMANOVA test. #—*p* < 0.05 and ##—*p* < 0.01 to compare with “Intact” mice, PERMANOVA test. ND—not determined.

The assessment of *E. faecalis* colonies number by PERMANOVA factor analysis revealed significant changes due to recovery time (control mice without antibiotics treatment, and 2 and 3 weeks after administration of probiotics) and recovery scheme (no treatment, gavage of *E. faecalis*, and gavage of *L. johnsonii*) (PERMANOVA F(2.24) = 6.36 *p* = 0.004; F(2.24) = 5.62 *p* = 0.002). After two weeks of probiotic administration, it was observed that the CFU of *E. faecalis* in all groups had already recovered to the rate of intact mice. A high number of *E. faecalis* CFU was maintained in the group receiving *E. faecalis* in the third week of the experiment (Figure 1A). Interestingly, after 3 weeks of *L. johnsonii* treatment, the CFU quantity of *E. faecalis* decreased significantly in comparison to the number of bacteria in the second time point. Additionally, when compared to other groups of mice, the CFU number of *E. faecalis* in the group receiving *L. johnsonii* was significantly lower (Figure 1A).

Similar to the microbiological bacterial counts, qPCR analysis of *E. faecalis* DNA level confirmed recovery by the second week in all groups, although the variation was high (Figure 1B). Three weeks after the start of the experiment, the amount of *E. faecalis* DNA was significantly lower in the group receiving *L. johnsonii* in comparison to other groups that confirmed the result of alive bacteria measurement by culture method (Figure 1A).

As for *L. johnsonii* amount changes, PERMANOVA factor analysis did not reveal significant effects of recovery period or recovery scheme on the CFU number (PERMANOVA F (2.24) = 0.09 *p* = 0.88; F(2.24) = 0.004 *p* = 0.99). In all groups, starting from the second week, the number of CFU was restored to the level of intact mice (Figure 1C). However, the amount of *L. johnsonii* DNA at week 3 was lower in both *L. johnsonii-* and *E. faecalis*-fed groups compared to naïve mice (Figure 1D). *L. johnsonii* DNA amount in feces was higher in the group that received *L. johnsonii* compared to the group that was gavaged with *E. faecalis*.

Thus, in the case of *L. johnsonii*, the number of CFU three weeks after the start of the experiment did not differ between the groups receiving *L. johnsonii* and *E. faecalis*, whereas *L. johnsonii* DNA was significantly higher in the group that received *L. johnsonii* and lower in the group with *E. faecalis*. And when analyzed, both CFU and DNA of *E. faecalis* were always higher in the group that was replanted with *E. faecalis*. To confirm the putative effect of *L. johnsonii* on *E. faecalis* growth, we performed a correlation analysis of the relationship between CFU and *E. faecalis* DNA counts in the groups receiving *E. faecalis* and *L. johnsonii*. A significant positive correlation was found between CFU and the amount of *E. faecalis* DNA in the group that was supplemented with *E. faecalis* probiotics (Figure 1E). In the group receiving *L. johnsonii*, the correlation between the number of DNA and CFU of *E. faecalis* bacteria was significantly negative (Figure 1F). Thus, in the group that was replanted with *L. johnsonii*, the greater the amount of *E. faecalis* DNA, the less live *E. faecalis* bacteria were detected in the feces of these animals, which confirms our previously observed negative effect of *L. johnsonii* on *E. faecalis* growth. Furthermore, there was no significant correlation between CFU and DNA of *L. johnsonii* in groups receiving *E. faecalis* and *L. johnsonii* (Figure 1G,H).

**Figure 2:**
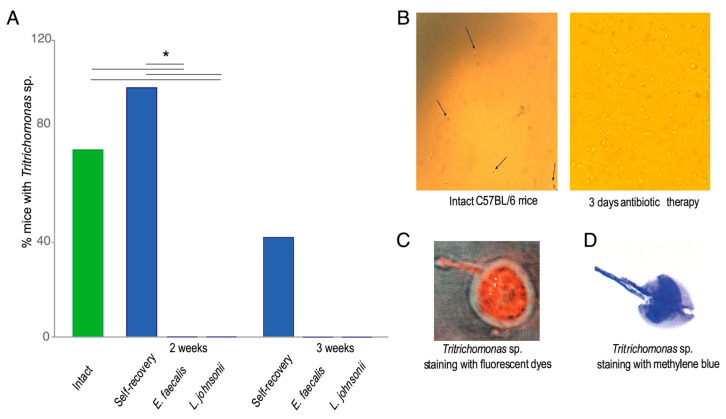
*Tritrichomonas* sp. contamination in mice before and after antibiotic treatment. (**A**). Percentage of *Tritrichomonas* sp. in mice before and after two weeks of antibiotic administration followed by gavage of probiotic bacteria *E. faecalis* and *L. johnsonii*. *—*p* < 0.05, Fisher’s exact test. (**B**)**.** Increase in *Tritrichomonas* sp. (indicated by arrows) number in mouse feces after 3 days of antibiotic treatment. (**C**). Staining of *Tritrichomonas* sp. with fluorescent dye CellTracker deep red. (**D**). Staining a smear of *Tritrichomonas* sp. with methylene blue.

During the microbiological analysis of bacterial populations in laboratory mice feces in steady state, *Tritrichomonas* sp. infection was found (Supplementary Video S1). Consistent with our previous publication, as well as data from other researchers, the presence of the protozoa itself did not influence the phenotypic characteristics of the histological profile [5,8]. Some changes typical for *Muc2*^−/−^ mice, such as crypt elongation, were observed in both control and experimental groups of mice [17]. In the group of intact mice, 76% of animals had microscopically detected protozoan infection. After the course of antibiotics followed by a two-week recovery period in the group without probiotics gavage, *Tritrichomonas* sp. was detected in all animals, while one week later, the number of mice with protozoa decreased to 40%. Interestingly, mice receiving *L. johnsonii* or *E. faecalis* did not show any protozoan infection by microscopic method throughout the probiotic treatment period (Figure 2A). In addition, antibiotic treatment for three days caused a considerable increase in *Tritrichomonas* sp. amount (Figure 2B–D).

## 3. Discussion

When using a variety of probiotic preparations to correct intestinal microbiota disorders, it is necessary to take into account the mutual influence of microorganisms. In our work, we studied the effects of two potential probiotic bacteria, *L. johnsonii* and *E. faecalis*, on its recovery in *Muc2*^−/−^ deficient mice after antibiotic administration. We showed that two weeks after antibiotics withdrawal and gavage of *L. johnsonii* or *E. faecalis*, there was no reciprocal effect of either *L. johnsonii* on the recovery of *E. faecalis* or vice versa. The amount of *L. johnsonii* and *E. faecalis* was comparable to that of intact animals. However, one week later, the effect of *L. johnsonii* on the number of *E. faecalis* was detected. In this group, there was a significant decrease in *E. faecalis* amount when mice were gavaged with *L. johnsonii*. This may be an indication of the negative effect of the actively growing population of *L. johnsonii* on the growth of *E. faecalis*. At the same time, the *E. faecalis* treatment had no effect on the growth of *L. johnsonii*. In the control group, without probiotic administration after antibiotic application, effective growth of *L. johnsonii* but not *E. faecalis* was observed. Thus, the recovery of *L. johnsonii* occurs quite efficiently, even without additional supplementation of *L. johnsonii*. It is known that some bacteria are able to compete with other bacteria from the same niche for metabolites and attachment sites. Such bacterial antagonism can be mediated via the production of organic acids, especially short-chain fatty acids, or by the production of bacteriocins [18]. Bacteriocins are a heterogeneous group of antimicrobial peptides with molecular masses between 2 and 10 kDa, which are synthesized by bacterial ribosomes and have activity against closely related bacterial strains. Lactobacilli secrete a number of bacteriocins: lactocins B, F, J, M, lactobrevin, plantaricin, etc. [19]. Some previous studies demonstrated that bacteriocin production can also be significantly induced by co-culture with specific bacteria. When *L. plantarum* was co-cultured with *E. faecium* or *Pediococcus pentosaceus*, bacteriocin activity was significantly increased and could inhibit the growth of bacteria belonging to *Bacillus*, *Enterococcus*, *Lactococcus*, and other genera [20,21]. Thus, it can be suggested that *L. johnsonii* suppresses the growth of *E. faecalis* and other bacteria, leading to a reduction in the diversity of microbiota. It is interesting to note that in the work of Ermolenko et al. [22], it was shown that in rats after a 3-day course of antibiotic therapy, *Enterococcus* spp. could recover even when receiving *L. fermentum*. However, the scheme of the experiment was different, as well as the bacterial strains.

Often, only one approach is used in the study of microbiota recovery. For example, in a study on the recovery efficiency of *L. johnsonii* and *E. coli* after fecal transplantation from healthy donors to mice, only the CFU counting method was used [23]. However, it is often hard to choose optimal conditions for culture as there are quite many fastidious bacteria, while some bacteria cannot be cultured in vitro. At the same time, many authors use the PCR method to evaluate the efficiency of microbiota recovery under different types of exposure [24,25,26]. The PCR assay is a very effective method for the assessment of bacterial diversity, including those that are difficult to culture. However, by PCR it is hard to discriminate between the presence of alive or dead bacteria. In our experiments, we used two methods to estimate the number of bacteria isolated from mouse feces: real-time qPCR analysis and microbiological analysis. Thus, both CFU and DNA amount of *E. faecalis* were always higher in the group that was administered with *E. faecalis* and the data of PCR analysis and microbiological analysis of *E. faecalis* are consistent. At the same time, some data obtained when analyzing the efficiency of probiotic bacteria recovery by PCR did not coincide with the data from microbiological analysis. Thus, in the case of *L. johnsonii*, the number of CFU three weeks after probiotics administration following antibiotic therapy did not differ between the groups receiving *L. johnsonii* and *E. faecalis*, whereas *L. johnsonii* DNA was significantly higher in the group that received *L. johnsonii* and lower in the group with *E. faecalis* gavage. This may be an indication that PCR analysis takes into account, among other things, the DNA of the bacteria that died during replanting. It should also be noted that when determining the amount of bacterial DNA by PCR after two weeks of bacterial replanting, there was a significant scatter in the data, and no reliable difference between the groups was found. Thus, it is necessary to carry out a comprehensive evaluation using different methods in order to obtain a more accurate conclusion of what is actually happening in the gut microbiota community.

When using antibiotics not only for experimental studies but also for therapeutic purposes, it is necessary to take into account that in addition to the antibacterial effect, such treatment may also influence other microbes, such as fungi and protozoa. For example, it was shown that in animals with a disturbed mucosal layer antibiotic treatment enhanced protozoa growth, which led to the death of animals [5]. The authors showed that the increase in *Tritrichomonas* sp. due to the reduction in bacterial community in *Muc2*^−/−^ mice was a key negative factor for the increased mortality rate. Therefore, it is necessary to consider the presence of non-bacterial components of the microbiota when antibiotics are used. Protozoa infection is difficult to diagnose, but as our data have shown, the reduction in bacterial content after antibiotic treatment allows for the effective detection of protozoa. It is possible that this approach may be useful for the diagnosis of other non-bacterial infections. More experiments are needed to better utilize this approach for the diagnosis of non-bacterial infections.

Our results showed that the application of *L. johnsonii* and *E. faecalis* promotes the elimination of *Tritrichomonas* sp. in the feces of *Muc2*^−/−^ mice. Possibly, this effect may be related to the cell wall polysaccharides of the probiotic bacteria. It has previously been shown that the application of poly- and monosaccharides eliminates protozoa in mice [5,27,28]. However, the mechanism of elimination is not yet fully understood. It is necessary to search for new approaches for antiprotozoal therapy as well as to emphasize the high probability of protozoan infections in patients with IBD.

## 4. Materials and Methods

### 4.1. Animals and Housing Conditions

Eight-week-old *Muc2*^−/−^ mice were kept in individually ventilated cages (Optimice, Animal Care Systems, Centennial, CO, USA) with constant access to food and water at 22–25 °C with a 14 h:10 h light-dark cycle (light phase from 1:00 to 15:00). All animals received a standard rodent diet (R-22, BioPro, Novosibirsk, Russia) and purified drinking water.

### 4.2. Experimental Design

**Figure 3:**
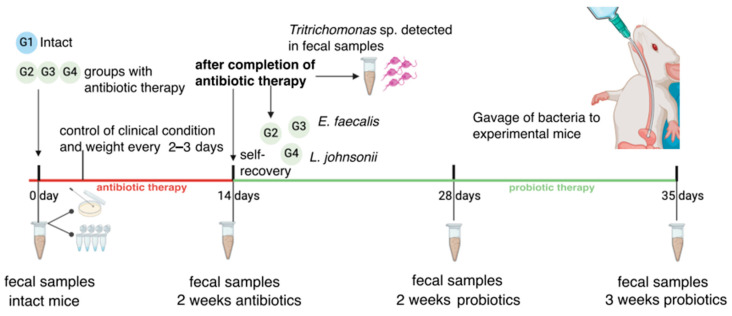
Schematic presentation of the experiment.

Mice were divided into 4 groups (5 animals/group): one group received no antibiotics (control group), and the other 3 experimental groups received an antibiotics cocktail for 14 days to suppress the growth of intestinal microbiota. The antibiotic cocktail was prepared in normal drinking water with the addition of 0.5 g/L gentamicin, 0.5 g/L ampicillin, 1 g/L vancomycin, and 0.5 g/L metronidazole. The antibiotics solution was prepared one day before administration to mice and stored at 4 °C. The weight of the mice and the clinical condition of the animals were monitored every 2–3 days during antibiotic therapy. After completion of antibiotics therapy, two experimental groups received either a solution of *L. johnsonii* (10^8^ CFU/mouse) or *E. faecalis* (10^8^ CFU/mouse) via oral gavage three times a week for three weeks. For another experimental group of mice, the antibiotics cocktail was substituted with normal drinking water without probiotics addition. Fecal samples were collected before the introduction of *L. johnsonii* or *E. faecalis*, 14 and 21 days after the bacterial gavage started. Fresh fecal samples were used to determine the number of viable *L. johnsonii* and *E. faecalis* bacteria. Samples for determination of bacterial DNA by real-time PCR were stored at −70 °C. The experimental design is shown in Figure 3.

All procedures were performed in accordance with the European Convention for the Protection of Vertebrate Animals Used for Experimental and Other Scientific Purposes and were approved by the local ethical committee of the Scientific Research Institute of Neurosciences and Medicine.

### 4.3. Isolation of L. johnsonii and E. faecalis Cultures

*L. johnsonii* and *E. faecalis* strains were isolated from mouse fecal samples. For this purpose, about 100 mg of intestinal content was grounded in 1000 µL of sterile PBS, and the resulting suspension was applied to the nutrient media-containing plates (HiMedia, Mumbai, India) for isolation of *Lactobacillus* and *Enterococcus* agar (TU 9398-110-78095326-2010, Obolensk, Russia) for isolation of *E. faecalis*. Petri dishes inoculated with bacteria from feces were incubated at 37 °C for at least two days under static aerobic and microaerobic conditions (5% CO_2_) using an anaerobic jar and CampyGen gas packs (Thermo Scientific, Waltham, WA, USA). Colonies grown on the plates were repeatedly inoculated to obtain pure cultures. The purity of isolated cultures was confirmed by seeding on CHROMagar™ Orientation (CHROMagar, Saint-Denis, France), microscopic examination, and 16S rRNA sequencing.

### 4.4. Culturing L. johnsonii and E. faecalis for Bacterial Colonization of Mouse Intestine

*L. johnsonii* and *E. faecalis* were inoculated using a bacteriological loop onto Petri dishes with MRS agar (HiMedia) and incubated overnight at +37 °C. Transfer to liquid medium was ensured by inoculation of 5 mL MRS broth. *L. johnsonii* were incubated microaerobically with 5% CO_2_, *E. faecalis* were incubated aerobically at +37 °C for 48 h. The bacterial titer in CFU/mL was determined in the obtained broths and further used for bacterial colonization of the intestine of mice.

### 4.5. Quantification of L. johnsonii and E. faecalis Cultures

The number of viable *L. johnsonii* and *E. faecalis* bacteria was determined in mouse feces using the drop plate method [29]. Fecal samples were dissolved in sterile PBS, and 10 µL of each serial dilution was seeded three times as a drop plate onto MRS agar (HiMedia) for the detection of *L. johnsonii* and onto enterococcagar for detection of *E. faecalis*. The seeded Petri dishes with MRS agar for detection of *L. johnsonii* were incubated at +37 °C for at least two days at static microaerobic (5% CO_2_) conditions. Petri dishes with enterococcagar to detect *E. faecalis* were incubated in a thermostat under aerobic conditions at +37 °C for two days. The colonies of each drop were counted, averaged, and multiplied by the dilution factor, and, taking into account the weight of the fecal sample, the CFU in 1 g of feces was determined. Colonies that showed typical morphology when growing on MRS and enterococcagar differential media were counted. Their belonging to one or another species was confirmed by PCR reaction and light microscopy.

### 4.6. Isolation of Bacterial DNA from Mice Feces

Isolation of DNA was conducted by DU-250 kit (Biolabmix, Novosibirsk, Russia) according to the protocol with modifications. Briefly, the fecal pellet was smashed in a lysis buffer, heated to 90 °C for 15 min, and centrifuged at 10,000 g for 3 min. After the addition of Proteinase K, the supernatant was heated to 55 °C for 20 min. Then, an equal amount of 95% ethanol was added, and the solution was transferred to the column. Following several washes, DNA was eluted, and its concentration was measured by NanoDrop2000 (Thermo Scientific, Waltham, WA, USA). An amount of 30 ng of DNA was used per reaction.

### 4.7. Determination of Bacterial DNA in Feces by Quantitative Real-Time PCR

The number of bacteria in feces was estimated by the amount of bacterial DNA of the 16S ribosomal RNA (rRNA) gene normalized to the DNA of the 28S rRNA gene of *Mus musculus*. The DNA-conserved region of the 16S rRNA gene was determined using primers to the 16S rRNA gene region of the corresponding group of bacteria. To determine the total number of bacteria, primers to the conserved region of the bacterial 16S rRNA gene were used. The amount of DNA of the 28S rRNA gene of *M. musculus* was determined using primer oligonucleotides (BIOSSET, Novosibirsk, Russia) (Table 2).

All primer sequences were selected using Primer-BLAST [30] and Unipro UGENE [31] programs. The reaction mixture (20 µL) contained BioMaster HS qPCR SYBR Blue 1× (Biolabmix), the corresponding primers (300 nM each), and DNA isolated from feces. PCR was performed in a CFX96 TouchTM Deep Well Real-Time PCR Detection System (Bio-Rad Laboratories Ltd, Hercules, CA, USA). DNA was denatured for 5 min at 95 °C; then 40 cycles were performed: denaturation—95 °C, 15 s; primer annealing—62 °C, 25 s; elongation—72 °C, 25 s.

Normalization of bacterial DNA to mouse (*M. musculus*) DNA was performed using the formula: 2^(Ct of the 28S rRNA gene of *M. musculus*—Ct of the 16S rRNA gene of *Bacteria*) for specific bacteria by the formula: 2^(Ct gene 16S rRNA *Bacteria*—Ct gene 16S rRNA specific to a particular bacterial community), where Ct is the cycle corresponding to the threshold luminescence level of the PCR product. Bacterial DNA levels in *Muc2*^−/−^ mice were expressed in conventional units.

### 4.8. Isolation of Tritrichomonas sp. Cultures from the Cecum of Mice

Cecum content was analyzed in a live drop, and active forms of *Tritrichomonas* sp. were detected (Supplementary Video S1). For isolation of the Protozoa from the cecum, the entire contents were placed in 25 mL of cold PBS after being carefully filtered (70 µm) and then centrifuged at 1200 rpm (+4 °C) for 5 min and washed twice with cold PBS. The washed samples were then applied to a gradient (Percoll 40%/80%) and centrifuged at 1000× *g* for 20 min. Interphase was then collected and washed twice with cold PBS. Then, aliquots were made for subsequent staining.

To increase the number of protozoa in the intestines, animals were administered with antibiotics in drinking water for 3 days. Antibiotics mixture contained clarithromycin (0.2 mg/mL) and amoxicillin (0.6 mg/mL) in accordance with the previously used protocol [5].

### 4.9. Staining of Tritrichomonas sp. with Fluorescent Dyes

CellTracker Deep Red Dye (Invitrogen, Waltham, WA, USA) and Hoechst fluorescent dye (Invitrogen, USA) were used to stain the protozoa. To 1 mL aliquots of cecum isolates, 10 µL (1 mM) each of CellTracker Deep Red Dye and Hoechst solution was added. The samples were then incubated for 40 min at +37 °C. After incubation, samples were placed on ice for 15 min and further centrifuged at 1000 rpm for 5 min. After washing with cold PBS, samples were analyzed using a fluorescence microscope (Carl Zeiss, Oberkochen, Germany).

### 4.10. Staining of Tritrichomonas sp. with Methylene Blue

For the preparation of smears, a drop of PBS containing *Tritrichomonas* sp. was applied on a slide and dried at room temperature. Then, the smear was fixed with methanol for 5 min. After fixation, the smear was placed in a bath with methylene blue solution for 5 min and then washed with distilled water. The smear was examined on a light microscope (Lomo, Saint Petersburg, Russia) at 10× and 40× objective lens magnification.

### 4.11. Statistics

Data in the text are presented as mean ± standard deviation. Graphics present data as a scatter diagram. Statistical processing of the data was performed using IBM SPSS Statistic software (version 23). Nonparametric PERMANOVA statistical processing methods were used to analyze samples not described by normal distribution. Comparison between the two groups for samples with normal distribution was performed using Student’s *t*-test and for samples without normal distribution using the Mann–Whitney test. Fisher’s exact test was used to compare the groups in which the trait was detected or not.

## 5. Conclusions

In this study, we conducted experiments on particular bacteria restoration after the administration of an antibiotic cocktail. Namely, we used *L. johnsonii* and *E. faecalis* as probiotic therapy, and the amount of these bacteria was assessed by two methods—microbiological culture and real-time qPCR. Two bacteria studied demonstrated various conduct of restoration. *L. johnsonii* was able to grow fast and recover to the initial amount, even without additional supplementation. While *E. faecalis* demonstrated the highest growth rate when providing the bacteria by oral gavage. At the same time, the amount of *E. faecalis* was decreased when treating mice with *L. johnsonii*, showing a comprehensive interaction of these bacteria. Furthermore, we showed that several methods are required to be used in combination for adequate evaluation as both microbiological analysis and qPCR have some restrictions. Finally, we observed that antibiotic treatment could induce the growth of *Tritrichomonas* sp., which could be inhibited by probiotic administration.

## Figures and Tables

**Table 1:** Sequence of the 16S rRNA gene of two bacterial species isolated from mice.

Species	Sequence 3’-5’	Compliance Percentage
*E. faecalis*	3’-ACATGCAAGTCGAACGCTTCTTTCCTCCCGAGTGCTTGCACTCATTTGGAAAGAGGAGTGGCGGACGGGTGAGTAACACGTGGG-5’ 3’-TAACCTACCCATCAGAGGGGGATAACACTTGGAAACAGGTGCTAATACCGCATAACAGTTTATGCCGCATGGCATAAGAGTGAAAG-5’ 3’-GCGCTTTCGGGTGTCACTGATGGATGGACCCGCGGTGCATTAGCTAGTTGGTGAGGTAACGGCTCACCAAGGCCACGATGCATAG-5’ 3’-CCGACCTGAGAGGGTGATCGGCCACACTGGGACTGAGACACGGCCCAGACTCCTACGGGAGGCAGCAGTAGGGAATCTTCGGC-5’ 3’-AATGGACGAAAGTCTGACCGAGCAACGCCGCGTGAGTGAAGAAGGTTTTCGGATCGTAAAACTCTGTTGTTAGAGAAGAACAAG-5’ 3’-GACGTTAGTAACTGAACGTCCCCTGACGGTATCTAACCAGAAAGCCACGGCTAACTACGTGCCAGCAGCCGCGGTAATACGTAGG-5’ 3’-TGGCAAGCGTTGTCCGGATTTATTGGGCGTAAAGCGAGCGCAGGCGGTTTCTTAAGTCTGATGTGAAAGCCCCCCGGCTCAACC-5’ 3’-GGGGAGGGTCATTGGAAACTGGGAGACTTGAGTGCAGAAGAGGAGAGTGGAATTCCATGTGTAGCGGTGAAATGCGTAGATAT-5’ 3’-ATGGAGGAACACCAGTGGCGAAGGCGGCTCTCTGGTCTGTAACTGACGCTGAGGCTCGAAAGCGTGGGGAGCAAACAGGATTA-5’ 3’-GATACCCTGGTAGTCCACGCCGTAAACGATGAGTGCTAAGTGTTGGAGGGTTTCCGCCCTTCAGTGCTGCAGCAAACGCATTAAG-5’ 3’-CACTCCGCCTGGGGAGTACGACCGCAAGGTTGAAACTCAAAGGAATTGACGGGGGCCCGCACAAGCGGTGGAGCATGTGGTTT-5’ 3’-AATTCGAAGCAACGCGAAGAACCTTACCAGGTCTTGACATCCTTTGACCACTCTAGAGATAGAGCTTTCCCTTCGGGGACAAAGT-5’ 3’-GACAGGTGGTGCATGGTTGTCGTCAGCTCGTGTCGTGAGATGTTGGGTTAAGTCCCGCAACGAGCGCAACCCTTATTGTTAGTTG-5’ 3’-CCATCATTTAGTTGGGCACTCTAGCGAGACTGCCGGTGACAAACCGGAGGAAGGTGGGGATGACGTCAAATCATCATGCCCCTTA-5’ 3’-TGACCTGGGCTACACACGTGCTACAATGGGAAGTACAACGAGTCGCTAGACCGCGAGGTCATGCAAATCTTTTAAAGCTTCTCTCA-5’ 3’-GTTCGGATTGCAGGCTGCAACTCGCCTGCATGAAGCCGGAATCGCTAGTAATCGCGGATCAGCACGCCGCGGTGAATACGTTCCC-5’ 3’-GGGCCTTGTACACACCGCCCGTCACACCACGAGAGTTTGTAACACCCGAAGTCGGTGAGGTAACCTTTTTGGAGCCAGCCGCCTA-5’	99.86 similarity to *E. faecalis* strain according to the NCBI Blast database
*L. johnsonii*	3’-CTAAATGAAACTAGATACAAGCGAGCGGCGGACGGGTGAGTAACACGTGGGTAACCTGCCCAAGAGACTGGGATAACACCTGGA-5’ 3’-AACAGATGCTAATACCGGATAACAACACTAGACGCATGTCTAGGGTTTGAAAGATGGTTCTGCTATCACTCTTGGATGGACCTGCG-5’ 3’-GTGCATTAGCTAGTTGGTAAGGTAACGGCTTACCAAGGCAATGATGCATAGCCGAGTTGAGAGACTGATCGGCCACATTGGGACT-5’ 3’-GAGACACGGCCCAAACTCCTACGGGAGGCAGCAGTAGGGAATCTTCCACAATGGACGAAAGTCTGATGGAGCAACGCCGCGTG-5’ 3’-AGTGAAGAAGGGTTTCGGCTCGTAAAGCTCTGTTGGTAGTGAAGAAAGATAGAGGTAGTAACTGGCCTTTATTTGACGGTAATTA-5’ 3’-CTTAGAAAGTCACGGCTAACTACGTGCCAGCAGCCGCGGTAATACGTAGGTGGCAAGCGTTGTCCGGATTTATTGGGCGTAAAGC-5’ 3’-GAGTGCAGGCGGTTCAATAAGTCTGATGTGAAAGCCTTCGGCTCAACCGGAGAATTGCATCAGAAACTGTTGAACTTGAGTGCA-5’ 3’-GAAGAGGAGAGTGGAACTCCATGTGTAGCGGTGGAATGCGTAGATATATGGAAGAACACCAGTGGCGAAGGCGGCTCTCTGGT-5’ 3’-CTGCAACTGACGCTGAGGCTCGAAAGCATGGGTAGCGAACAGGATTAGATACCCTGGTAGTCCATGCCGTAAACGATGAGTGCT-5’ 3’-AAGTGTTGGGAGGTTTCCCGCCTCTCAGTGCTGCAGCTAACGCATTAAGCACTCCGCCTGGGGAGTACGACCGCAAGGTTGAA-5’ 3’-ACTCAAAGGAATTGACGGGGGCCCGCACAAGCGGTGGAGCATGTGGTTTAATTCGAAGCAACGCGAAGAACCTTACCAGGTC-5’ 3’-TTGACATCCAGTGCAAACCTAAGAGATTAGGTGTTCCCTTCGGGGACGCTGAGACAGGTGGTGCATGGCTGTCGTCAGCTCGT-5’ 3’-GTCGTGAGATGTTGGGTTAAGTCCCGCAACGAGCGCAACCCTTGTCATTAGTTGCCATCATTAAGTTGGGCACTCTAATGAGAC-5’ 3’-TGCCGGTGACAAACCGGAGGAAGGTGGGGATGACGTCAAGTCATCATGCCCCTTATGACCTGGGCTACACACGTGCTACAATG-5’ 3’-GACGGTACAACGAGAAGCGAACCTGCGAAGGCAAGCGGATCTCTTAAAGCCGTTCTCAGTTCGGACTGTAGGCTGCAACTCG-5’ 3’-CCTACACGAAGCTGGAATCGCTAGTAATCGCGGATCAGCACGCCGCGGTGAATACGTTCCCGGGCCTTGTACACACCGCCCGT-5’ 3’-CACACCATGAGAGTC-5’	99.86 similarity to *L. johnsonii* strain according to the NCBI Blast database

**Table 2:** Primer sequences.

Target	Primer Name	Primer Sequence 5’->3’
Bacterial 16S	16S F	TCCTACGGGAGGCAGCAG
	16S R	ATTACCGCGGCTGCTGG
Mouse 28S	28S F	CCTGGCGCTAAACCATTCGT
	28S R	AAAGCCCGCAGAGACAAACC
*L. johnsonii* 16S	Lac_john F Lac_john R	GATTAGGTGTTCCCTTCGGGG TCGCTTCTCGTTGTACCGTC
*E. faecalis* 16S	Ent_faecalis F Ent_faecalis R	GAACGTCCCCTGACGGTATC GTTTACGGCGTGGACTACCA

## Data Availability

The data presented in this study are available upon request from the corresponding author.

## Supplementary Materials

The following supporting information can be downloaded at https://www.mdpi.com/article/10.3390/ijms25105090/s1.
